# Targeting SARS-CoV-2 programmed −1 ribosomal frameshifting: structural dynamics and RNA-directed antiviral strategies

**DOI:** 10.1128/aac.00687-26

**Published:** 2026-08-03

**Authors:** Neha Jeena, Inshad Ali Khan

**Affiliations:** 1 Department of Microbiology, School of Life Sciences, Central University of Rajasthan 726899, Ajmer, Rajasthan, India; Samsung Medical Center Department of Infectious Diseases, Seoul, Republic of Korea

**Keywords:** programmed −1 ribosomal frameshifting, SARS-CoV-2 RNA pseudoknot, RNA-targeted therapeutics, conformational dynamics

## Abstract

Programmed −1 ribosomal frameshifting (−1 PRF) is a translational recoding mechanism used by many RNA viruses to regulate the expression of viral replication proteins. In coronaviruses, including SARS-CoV-2, −1 PRF is controlled by a conserved frameshift stimulation element containing a three-stemmed RNA pseudoknot located downstream of a slippery sequence. Studies have shown that conformational dynamics, mechanical stability, and structural variability of the pseudoknot influence ribosome pausing and frameshifting efficiency, identifying viral RNA structures as potential antiviral targets. This review outlines the structural organization, mechanistic basis, and conformational dynamics of viral frameshifting pseudoknots, with emphasis on the SARS-CoV-2 frameshift stimulation element. Advances in cryo-electron microscopy, single-molecule biophysics, molecular dynamics simulations, and computational modeling have identified multiple pseudoknot conformations involved in translational recoding and ribosome–RNA interactions. RNA-targeted therapeutic approaches used to suppress or modulate −1 PRF are also discussed, including small-molecule RNA binders, antisense oligonucleotides, peptide nucleic acids, and ribonuclease-targeting chimeras. These approaches act on distinct aspects of RNA structure, conformational flexibility, and stability to inhibit viral translation or promote selective RNA degradation. Major challenges include selective targeting of highly dynamic RNA structures, optimization of intracellular delivery, and minimizing off-target effects. Integration of structural biology, computational modeling, and RNA-targeted therapeutic strategies may support the development of next-generation antivirals targeting conserved viral RNA regulatory elements.

## INTRODUCTION

RNA viruses represent one of the major global health problems because of their ability to mutate, spread quickly, and adapt between different species (1). Current antiviral strategies against RNA viruses primarily include vaccines, inhibitors targeting viral enzymes, non-enzymatic viral proteins, host-directed therapies, emerging approaches directed against conserved viral RNA elements, and, in some cases, agents targeting viral lipids and glycans (2, 3). However, the rapid emergence of viral mutations and drug resistance has highlighted the need for alternative therapeutic approaches targeting conserved viral RNA elements (4). Programmed −1 ribosomal frameshifting (−1 PRF) represents a mechanistically attractive target that requires further antiviral validation (5).

In order to increase coding efficiency, RNA viruses use translational recoding strategies, such as −1 PRF, that allow the expression of several proteins through overlapping reading frames. In −1 PRF, the ribosome moves backward along the viral mRNA by one nucleotide, thus generating a new reading frame and allowing the expression of different viral proteins. The efficiency of −1 PRF is tightly regulated to maintain the proper stoichiometric balance between structural and replicase proteins. Any slight change in frameshifting efficiency may have a major impact on virus replication, thereby making −1 PRF a potential target for antiviral intervention that warrants further validation (6, 7).

Among the stimulatory RNA elements involved in −1 PRF, pseudoknots are the most abundant and physiologically relevant structures. Pseudoknots result from the formation of base pairs between the loops and complementary sequences at their distal end, resulting in the formation of small RNA tertiary structures held together by coaxial stacking and non-canonical base-pair interactions. Coronaviruses contain frameshifting elements arranged in a three-stem pseudoknot configuration, which provides high structural stability and is resistant to ribosome unwinding (8).

During translation, the formation of a pseudoknot on the translating mRNA causes mechanical obstruction, thus slowing down the ribosome, increasing the likelihood of ribosomal slippage at the slippery sequence upstream. −1 PRF is regulated by multiple factors, including RNA sequence, RNA structure, and ribosome activity. Moreover, recent findings have revealed that these RNA elements are dynamic structures, adding another layer of regulation to −1 PRF (9).

Though advances have been made in characterizing the structure of coronavirus pseudoknots and interactions with ribosomes, the connection between RNA dynamics and frameshifting efficiency is still unclear. Since pseudoknot conformations are dynamic and varied, designing antiviral drugs targeting RNA molecules to influence −1 PRF becomes an immense challenge (10).

This review provides an overview of the structural organization, mechanistic basis, and conformational dynamics of viral frameshift pseudoknots with particular emphasis on SARS-CoV-2. We further discuss emerging RNA-targeted therapeutic strategies aimed at modulating −1 PRF and highlight current challenges associated with targeting dynamic viral RNA structures.

## STRUCTURAL ORGANIZATION OF VIRAL FRAMESHIFTING PSEUDOKNOTS

**Fig 1:**
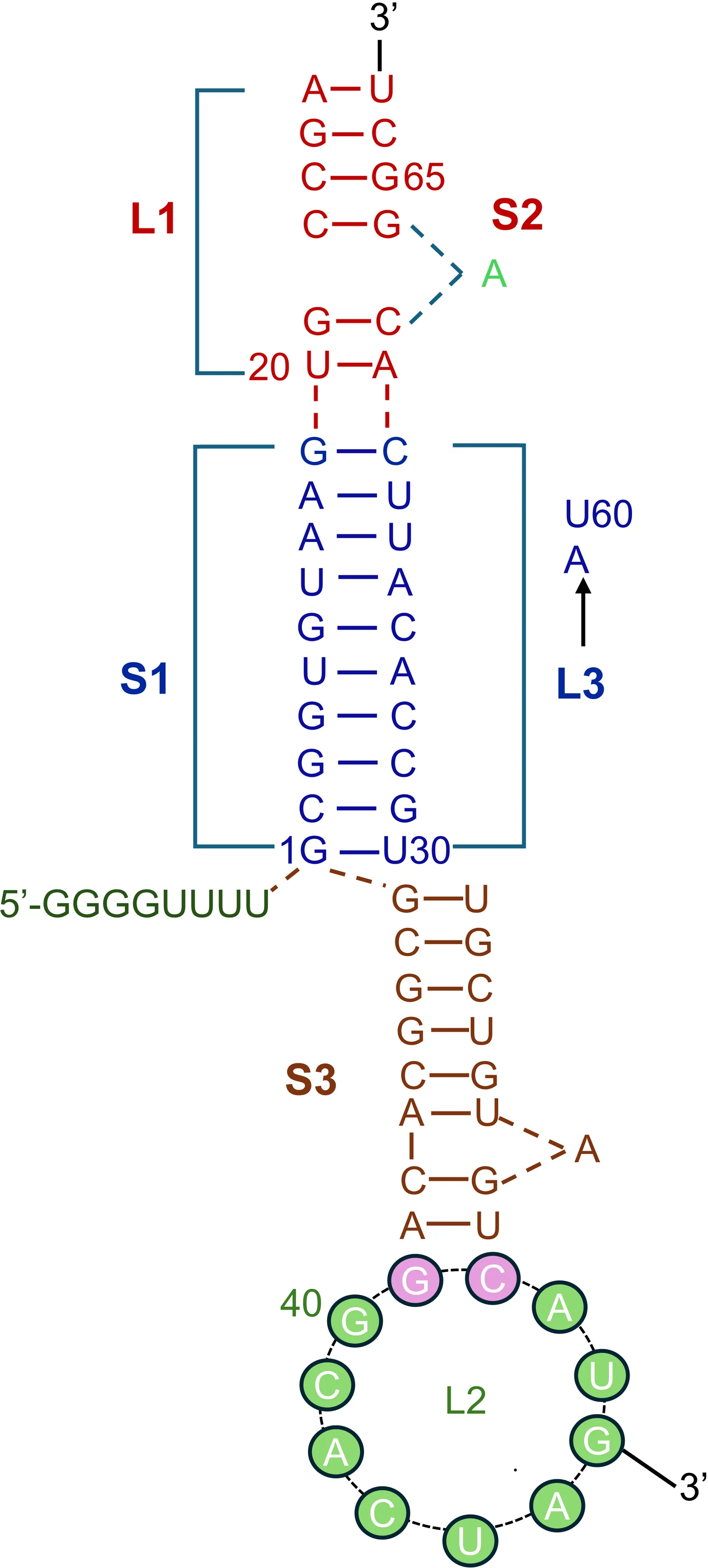
Secondary structure of the SARS-CoV-2 programmed −1 ribosomal frameshifting pseudoknot showing the three-stem architecture. Stem 1 (blue), Stem 2 (red), and Stem 3 (brown), along with Loop 1, Loop 2, and Loop 3, are indicated.

Coronavirus programmed −1 ribosomal frameshifting is stimulated by a compact H-type RNA pseudoknot located downstream of the slippery sequence. The canonical H-type pseudoknot is formed when nucleotides within a loop base pair with complementary downstream sequences, generating two helical stems (Stem 1 [S1] and Stem 2 [S2]) connected by Loop 1 (L1) and Loop 2 (L2) (11). In coronaviruses, including SARS-CoV-2, the frameshift-stimulatory element contains an additional Stem 3 (S3), connected through Loop 3 (L3), giving rise to the characteristic three-stemmed architecture shown in Fig. 1 (12).

The overall organization of the coronavirus pseudoknot is further stabilized by tertiary interactions. Stem 1 and Stem 2 adopt a coaxial arrangement, while Stem 3 associates with the pseudoknot core through loop–helix contacts, base triples, and other non-canonical RNA interactions. Together, these interactions maintain the compact architecture of the pseudoknot and contribute to its structural stability, which is essential for efficient programmed −1 ribosomal frameshifting (12, 13). Although the three-stemmed architecture is conserved, coronavirus pseudoknots can adopt multiple conformational states that influence their mechanical properties and frameshifting efficiency (14–17).

## MECHANISTIC BASIS OF −1 PRF

**Fig 2:**
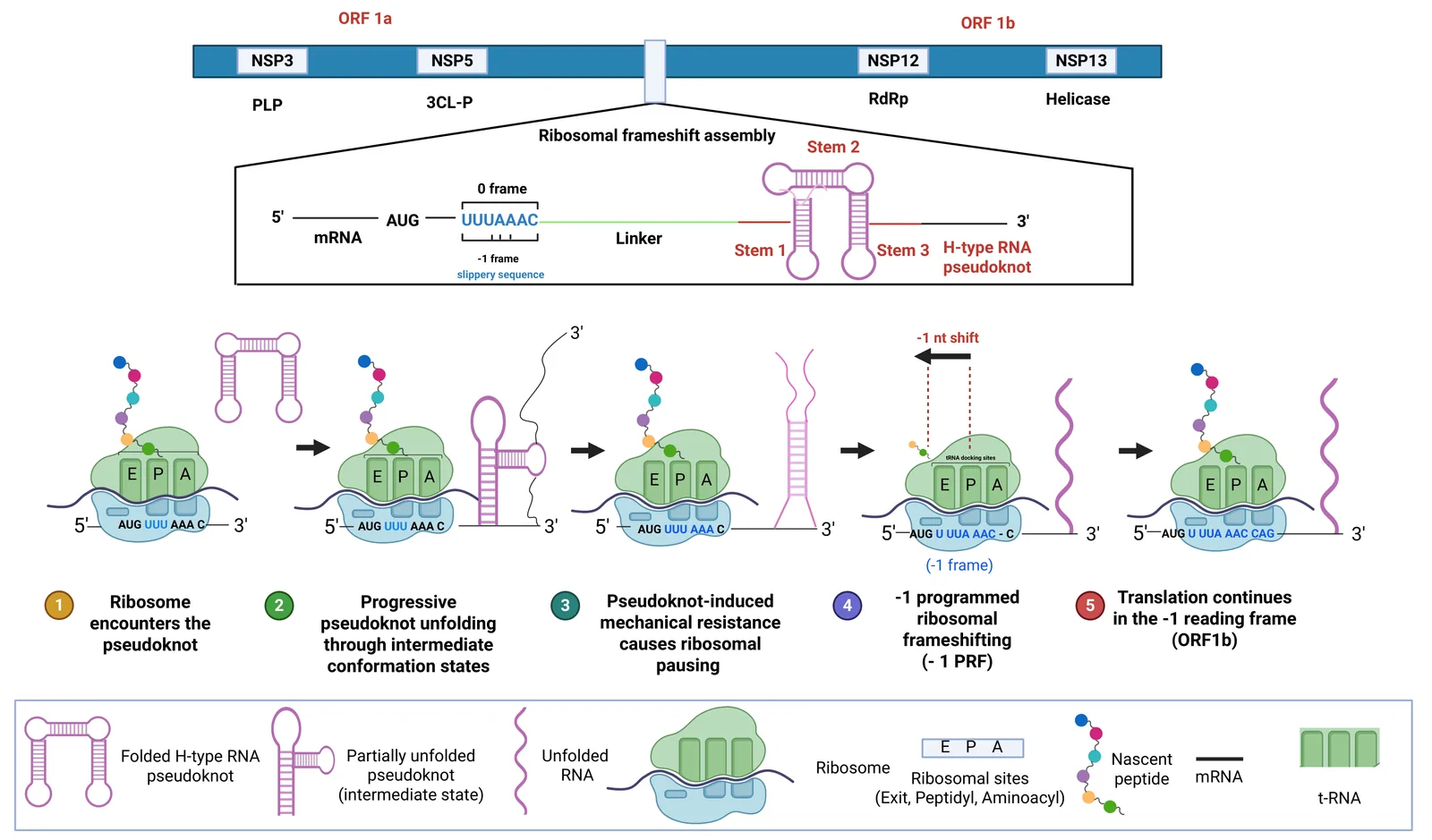
Mechanism of programmed −1 PRF in SARS-CoV-2. Schematic representation of the SARS-CoV-2 ORF1a/ORF1b frameshift region illustrating ribosomal progression toward the slippery sequence and the downstream RNA pseudoknot. Pseudoknot-induced mechanical resistance promotes ribosomal pausing and one-nucleotide backward slippage into the −1 reading frame. Subsequent pseudoknot unfolding allows translation to continue in ORF1b, resulting in the synthesis of viral replication proteins.

Programmed −1 ribosomal frameshifting depends on the coordinated interaction between specific RNA elements and ribosomal dynamics (18). A key element in this mechanism is the slippery sequence, which is characterized by the pattern UUUAAC and in which tRNAs placed at the ribosomal P and A sites shift backward by one nucleotide while maintaining codon-anticodon pairing (19, 20). This sequence is located upstream of the RNA pseudoknot and separated by a certain spacer region, which regulates the timing of ribosome-RNA interactions (21). Upon encountering the pseudoknot, the ribosome experiences mechanical obstruction, leading to ribosomal pausing and increasing the probability of tRNA slippage into the −1 frame (Fig. 2) (22).

In particular, the elongation factor-G becomes partially uncoordinated in relation to other ribosomal movements, thereby promoting ribosomal rearrangements associated with frameshifting. Ribosomal pausing alters tRNA dynamics and disrupts normal translocation, increasing the likelihood of tRNA realignment into an alternative reading frame. Structural studies further suggest that frameshifting occurs during the late stages of translocation when ribosomal movements such as ribosomal head swivel are disrupted by the downstream RNA pseudoknot (23).

## CONFORMATIONAL DYNAMICS OF FRAMESHIFTING PSEUDOKNOTS

**Fig 3:**
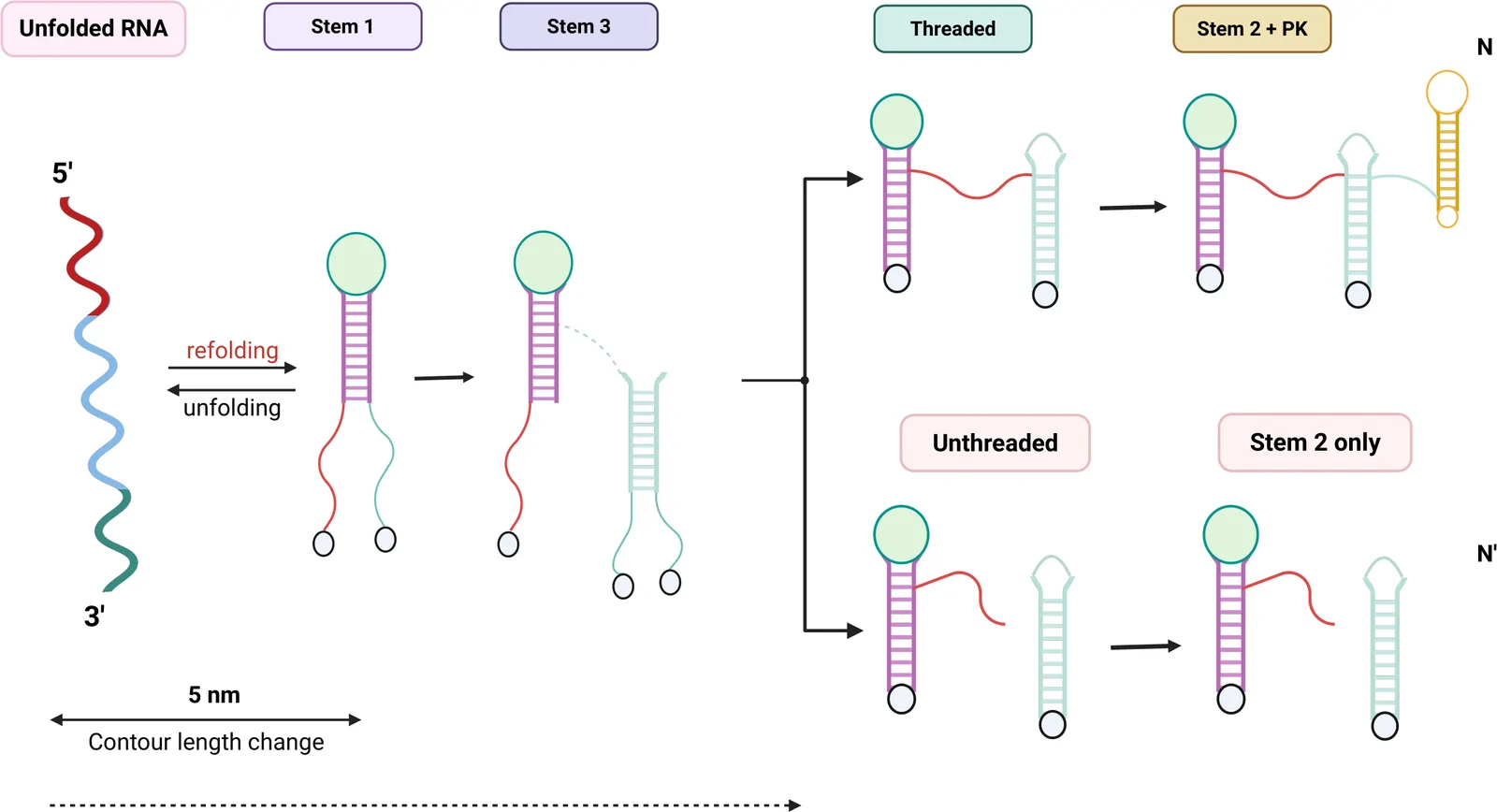
Conformational folding pathway of the SARS-CoV-2 −1 PRF pseudoknot. Sequential folding and unfolding of the three-stemmed pseudoknot showing intermediate folding states and the formation of threaded and unthreaded conformers.

Coronavirus frameshifting pseudoknots do not adopt a single rigid structure but exist as dynamic conformational ensembles that interconvert between multiple structural states. Single-molecule optical tweezer studies of the SARS-CoV-2 frameshift-stimulatory pseudoknot have shown that its unfolding proceeds through multiple intermediate states along a rugged energy landscape rather than through a simple two-state folding–unfolding process (Fig. 3) (24). These conformational transitions determine the mechanical resistance encountered by the translating ribosome and thereby influence programmed −1 ribosomal frameshifting efficiency (16, 17).

During translation, the ribosome encounters the downstream pseudoknot after decoding the slippery sequence and begins to unwind its helical stems. Instead of unfolding cooperatively, the pseudoknot dissociates through sequential intermediate states. Optical tweezer experiments indicate that Stem 1 forms first during folding, followed by Stem 3, whereas Stem 2 forms last to complete the three-stemmed structure. The position of the 5′ end during the final folding step determines whether the pseudoknot adopts a threaded or an unthreaded topology. The threaded conformer exhibits greater mechanical stability and offers higher resistance to ribosomal unwinding than the unthreaded conformer, resulting in prolonged ribosomal pausing and altered frameshifting efficiency (16, 24, 25).

Molecular dynamics (MD) simulations, together with cryo-electron microscopy (cryo-EM) and small-angle X-ray scattering-based structural analyses, have shown that the SARS-CoV-2 pseudoknot continuously samples multiple conformations rather than remaining in a single structural state (10, 15, 16). The relative stability of these conformations is influenced by RNA tertiary interactions, stem stability, sequence composition, and divalent ions such as Mg²^+^, which collectively affect the mechanical properties of the pseudoknot during translation. Mutations that restrict conformational flexibility reduce frameshifting efficiency, underscoring the functional importance of RNA structural plasticity (15).

The ability of coronavirus frameshifting pseudoknots to sample multiple conformations is therefore closely linked to their biological function. By modulating ribosomal pausing and the probability of frameshifting, conformational dynamics provide an additional level of translational regulation and represent an attractive target for RNA-directed antiviral strategies (17, 26).

## COMPUTATIONAL AND MOLECULAR DYNAMICS APPROACHES

Computational simulations and biophysics techniques have contributed significantly to the understanding of structural organization and conformational dynamics of viral frameshifting pseudoknots. Among these approaches, molecular dynamics simulations have been particularly valuable for capturing the conformational flexibility of RNA pseudoknots (27). MD simulations reveal dynamic structural changes, including helix bending, loop rearrangements, and disruptions of tertiary interactions that contribute to RNA structural flexibility (28). These conformational dynamics influence the response of the RNA pseudoknots to ribosome-mediated mechanical forces, thereby affecting frameshifting efficiency through changes in mechanical resistance and spacer tension (16).

Virtual screening approaches have also been applied based on structural information obtained from MD simulations (29). Using ensembles of RNA conformations obtained, computational approaches have helped identify ligands targeting transient or partially formed pockets within RNA pseudoknots, providing insight into RNA-targeted therapeutic approaches for modulating −1 PRF. However, further evaluation in physiologically relevant infection models is needed to determine whether these approaches can effectively inhibit viral replication. The use of machine learning to predict RNA-ligand interactions has increased in recent years (30). By training the computational models on known RNA-binding compounds, patterns associated with binding specificity and affinity can be identified, thereby facilitating hit identification and lead optimization (31). Coupling machine learning with conformational ensembles obtained from MD simulations further improves the prediction and design of ligand-binding modes for dynamic RNA structures (32).

Combining computational approaches with experimental techniques such as nuclear magnetic resonance (NMR) spectroscopy has also been critical in identifying novel RNA-ligand-binding pockets (33). In particular, NMR coupled with MD simulations has helped identify multiple ligand-binding pockets and characterize their dynamic behavior upon ligand interaction (34).

In addition, computational approaches have played an important role in optimizing nucleic acid-based therapeutic strategies, including peptide nucleic acids (PNAs) and antisense oligonucleotides (ASOs), by predicting structural changes associated with RNA-ligand interactions and improving target specificity (35).

Collectively, MD simulation, virtual screening, machine learning algorithms, and integrative structural approaches provide complementary strategies for understanding RNA conformational dynamics and accelerating the development of RNA-targeted antiviral ligands.

## RNA-TARGETED THERAPEUTIC APPROACHES

The structural complexity and conformational flexibility of viral RNA pseudoknots involved in −1 PRF provide multiple opportunities for therapeutic intervention. Since −1 PRF is governed by a finely tuned interplay between RNA structure, mechanics, and kinetics, even subtle perturbations in pseudoknot structure or conformational dynamics can significantly influence frameshifting efficiency. Consequently, RNA-targeted therapeutic strategies are being investigated to disrupt pseudoknot structure and dynamics, with the goal of modulating −1 PRF and evaluating their antiviral potential (36).

**Fig 4:**
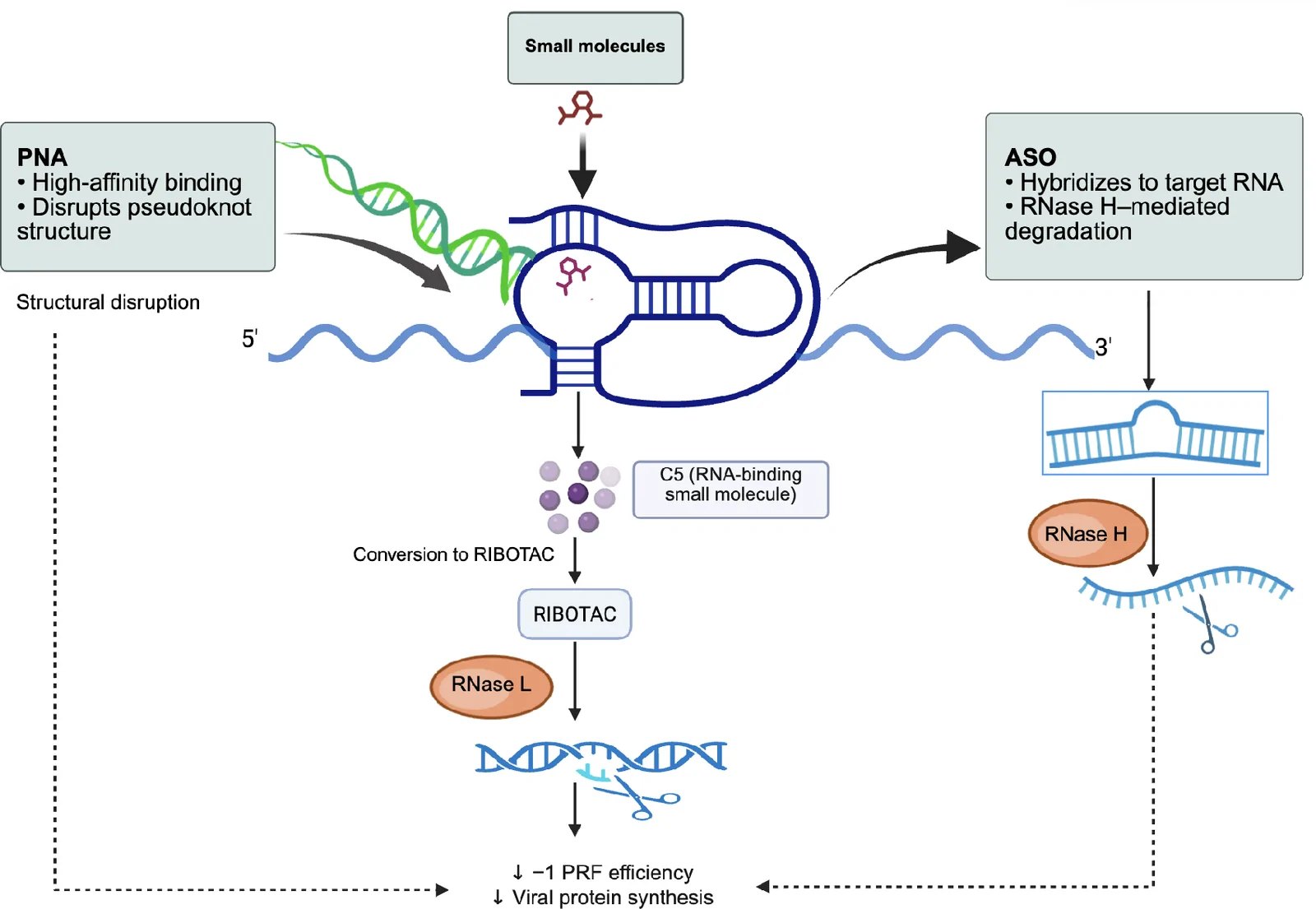
RNA-targeting strategies against the SARS-CoV-2 frameshifting pseudoknot. Schematic illustration of RNA-targeted therapeutic approaches to inhibit SARS-CoV-2 programmed −1 PRF. PNAs disrupt pseudoknot structure, small molecules modulate RNA conformational dynamics, ASOs induce RNase H-mediated cleavage, and C5-derived RIBOTACs promote RNase L-mediated viral RNA degradation, collectively reducing viral protein synthesis and replication.

Current RNA-targeted antiviral approaches include small molecules that modulate RNA pseudoknot conformations, oligonucleotide-based strategies such as antisense oligonucleotides and peptide nucleic acids, and RNA degradation methods, including ribonuclease-targeting chimeras (RIBOTACs) (Fig. 4). These approaches target distinct aspects of RNA structure, stability, and conformational dynamics and have been shown to modulate −1 PRF in biochemical, cellular, and limited antiviral model systems.

### Small-molecule RNA binders

Targeting viral frameshifting pseudoknots with small molecules represents an emerging approach for modulating −1 PRF. Structured RNA elements like pseudoknots present intricate three-dimensional architectures—helices, loops, junctions, and base-triple interactions—that create distinct ligand-binding pockets (37, 38). In coronavirus frameshifting elements, these pockets are often found at helix-junction interfaces and at sites of tertiary interactions that stabilize the compact three-stem H-type pseudoknot fold (25). Mechanistically, small molecules influence −1 PRF mainly by altering the pseudoknot’s structural integrity and conformational dynamics (38).

Efficient frameshifting relies on the RNA’s capacity to generate sufficient mechanical resistance against the ribosome during translocation, triggering translational pausing and promoting tRNA slippage at the upstream slippery sequence. When a ligand disrupts key tertiary interactions—such as base triples or coaxial stacking between stems—it can destabilize mechanically resistant conformations, shorten ribosomal pausing, and thereby decrease frameshifting efficiency (15). Conversely, excessive stabilization or rigidification of certain RNA regions may limit the flexibility needed to access intermediate states within the folding landscape, which are also crucial for optimal −1 PRF (39). Structural and biochemical studies provide direct evidence for these mechanisms. For example, aminoquinazoline derivatives bind the SARS-CoV-2 frameshifting element and modulate −1 PRF in cellular systems, likely by perturbing local RNA structure and flexibility (40). Small molecules targeting the pseudoknot alter its conformational dynamics and mechanical stability, thereby influencing frameshifting efficiency. These findings emphasize that ligand efficacy depends not only on binding affinity but also on the ability to modulate conformational dynamics and structural plasticity of the RNA pseudoknot (39).

A number of small-molecule inhibitors targeting coronavirus frameshifting pseudoknots have been validated using biochemical and cell-based assays. These compounds exhibit diverse mechanisms, including modulation of RNA conformational dynamics, direct binding to pseudoknot junctions, and interference with ribosome-RNA interactions. Importantly, several compounds have demonstrated functional inhibition of −1 PRF in dual-luciferase and cell-based assays (41, 42).

Representative examples of experimentally validated inhibitors are summarized in Table 1. In addition to these compounds, several small molecules uncovered through high-throughput screening approaches have been reported to modulate −1 PRF across multiple coronaviruses (43). Notably, fluoroquinolone-based compounds such as merafloxacin inhibit coronavirus frameshifting elements in experimental systems, supporting further investigation of conserved RNA-targeting strategies. These findings highlight the importance of integrating structural and functional screening to identify ligands capable of selectively targeting dynamic RNA elements (44).

**TABLE 1:** Experimentally validated small molecules targeting the coronavirus RNA pseudoknot to suppress programmed −1 PRF and viral replication

Compound	Target	Assay	Effect	Mechanism of action	Reference
Merafloxacin	SARS-CoV-2 frameshift stimulation element	Dual-luciferase reporter assay; viral plaque assay	IC_50_ = 20 µM (−1 PRF inhibition); EC_50_ = 2.6 µM (SARS-CoV-2 replication); CC_50_ >37 µM (Vero E6 cells)	Modulates pseudoknot conformational dynamics	(41)
Carrimycin	SARS-CoV-2 frameshift stimulation element	Dual-luciferase reporter assay; biochemical binding assay	EC_50_ = 6.29–14.14 μg/mL; CC_50_ = 28.28 µg/mL.	Direct RNA binding	(45)
Aminoquinazoline derivatives (Compound 13)	SARS-CoV-2 frameshift stimulation element	Dual-luciferase reporter assay	*K*_*D*_ = 60 ± 6 µM; ~50% inhibition of −1 PRF at 50 µM.	RNA-targeting ligands affecting structure	(40)
C5 (and derivatives)	SARS-CoV-2 frameshift stimulation element	Cell-based assay; biochemical assay	*K*_*D*_ = 11 ± 3.8 nM; ~25% reduction in −1 PRF at 2 µM	RNA binding; RIBOTAC precursor	(46)
Ivacaftor	SARS-CoV-2 frameshift stimulation element	Dual-luciferase reporter assay	96% reduction in −1 PRF (luciferase reporter assay).	Functional −1 PRF inhibitor	(47)
MTDB	SARS-CoV frameshifting pseudoknot (model for SARS-CoV-2)	Dual-luciferase reporter assay	~60% reduction in −1 PRF activity (dual-luciferase assay).	Junction-targeting RNA binder	(5)
Nafamostat	Coronavirus frameshifting pseudoknot	Dual-luciferase reporter assay	IC_50_ = 0.5 ± 0.4 µM; ~50% reduction in SARS-CoV-2 −1 PRF at 20 µM.	Functional −1 PRF inhibitor	(43)
Valnemulin	SARS-CoV-2 frameshift stimulation element	Dual-luciferase reporter assay	IC_50_ = 0.04 ± 0.03 µM; ~35% reduction in SARS-CoV-2 −1 PRF at 20 µM.	Ribosome-associated modulation of frameshifting	(43)
KCB261770	Coronavirus frameshifting pseudoknot (MERS-CoV, SARS-CoV, and SARS-CoV-2)	Dual-luciferase reporter assay	IC_50_ = 0.07 µM; CC_50_ = 18.5 µM; 91.6% inhibition of MERS-CoV −1 PRF (5 µM); SARS-CoV-2 IC_50_ ≈ 0.54 µM.	Broad-spectrum frameshifting inhibitor	(42)
Abemaciclib	SARS-CoV-2 frameshift stimulation element	Dual-luciferase reporter assay; cell-based assay	IC_50_ = 0.6 ± 0.2 µM; ~30% reduction in SARS-CoV-2 −1 PRF at 20 µM.	Moderate inhibition of −1 PRF	(43)
Palbociclib	SARS-CoV-2 frameshift stimulation element	Dual-luciferase reporter assay; cell-based assay	IC_50_ = 0.6 ± 0.3 µM; ~30% reduction in SARS-CoV-2 −1 PRF at 20 µM.	Moderate inhibition of −1 PRF	(43)
Geneticin (G418) and analogs	SARS-CoV-2 frameshift stimulation element	Dual-luciferase reporter assay; viral replication assay	EC_50_ = 25.7–129 µM (SARS-CoV-2 variants); CC_50_ = 934–3,951 µM; reduced −1 PRF efficiency.	Inhibits −1 PRF and viral replication	(48)

The dynamic nature of frameshifting pseudoknots adds an extra layer of complexity to small-molecule targeting. Single-molecule and computational studies reveal that these RNA elements exist as ensembles of interconverting conformations, including threaded and unthreaded topologies with distinct mechanical properties (25).

Small molecules may preferentially bind to specific states, shifting the ensemble’s equilibrium. Stabilizing less compact or mechanically weaker conformations lowers the force required for ribosomal unwinding, reducing pausing and −1 PRF efficiency. Conversely, trapping the RNA in a rigid but non-productive conformation can also hinder frameshifting by blocking essential conformational transitions during translation. This conformation-selective targeting represents an emerging paradigm in RNA-directed drug discovery (44).

In addition to altering RNA structure and dynamics, an important consideration is the interplay between ligand binding and RNA-ribosome interactions. Cryo-EM studies show the pseudoknot lodging at the mRNA entry channel of the ribosome, where it directly resists helicase activity during elongation (49).

Small molecules that alter pseudoknot geometry or positioning may therefore influence how the RNA engages with the ribosome, modulating the mechanical tension transmitted to the slippery site and ultimately affecting frameshifting efficiency. In this way, small-molecule binding can indirectly regulate ribosome dynamics and translocation kinetics (24).

Despite these advances, several challenges limit the development of small-molecule therapeutics targeting −1 PRF. Achieving high specificity is difficult due to the structural similarity of RNA motifs and the limited chemical diversity of RNA-binding pockets. In addition, the dynamic and flexible nature of RNA complicates structure-based drug design, as ligands must effectively engage transient or metastable conformations within the folding landscape. Off-target interactions with host RNAs remain a concern, underscoring the need for highly selective compounds (50). To overcome these limitations, recent approaches combine high-resolution structural data with MD simulations and ensemble-based screening strategies (51). These methods aim to identify ligand-binding sites that are both structurally unique and functionally important for frameshifting. Such approaches may facilitate the development of ligands with improved specificity and biological activity. By targeting conformational hotspots that regulate mechanical resistance and ribosome pausing, future studies may facilitate the design of small molecules with improved specificity that can subsequently be evaluated for antiviral activity (52).

Overall, targeting frameshifting pseudoknots with small molecules represents an emerging strategy to modulate viral translation by reshaping RNA structure and dynamics. Rather than acting merely as inhibitors, these compounds act as modulators of RNA conformational landscapes, highlighting the need for integrated structural biology, biophysics, and computational modeling in developing RNA-targeted antivirals (53).

Future directions are likely to emphasize ensemble-based ligand design and structure-guided optimization. Given the dynamic nature of frameshifting pseudoknots, addressing transient or intermediate conformational states may improve both specificity and efficacy. Advances in cryo-electron microscopy and molecular dynamics simulations are expected to facilitate the identification of such conformational hotspots and support the discovery and preclinical optimization of RNA-targeted therapeutic candidates (54, 55).

### Antisense oligonucleotides

ASOs provide a sequence-based approach that targets the SARS-CoV-2 frameshift-stimulating element. Unlike small molecules that rely on dynamic RNA structures to detect potential druggable pockets, ASOs depend on Watson-Crick base pairing to recognize the necessary structural components present in the frameshift-stimulating element pseudoknot structure. These structural components comprise indispensable stems, loops, and spacers necessary for the initiation of −1 PRF (56, 57). The inhibition of −1 PRF is achieved by disrupting the H-type structure of the pseudoknot through physical obstruction of the structural modifications necessary for the ribosome to stop at the slippery site and undergo frameshifting. This leads to reduced −1 PRF and thus inhibition of the downstream protein translation of the key viral replication proteins, especially the RNA-dependent RNA polymerase located in ORF1b (58).

#### Chemical engineering and hybridization potency

The effectiveness of ASOs as an intracellular therapy depends on chemical modifications that protect ASOs against nuclease degradation and increase their ability to bind highly structured RNAs. Locked nucleic acids (LNA) are usually used for modification of ASOs that target the frameshifting element. Due to their ability to lock the ribose sugar to the C3′-endo conformation, LNA modifications lead to significant increases in melting temperature (Tm) and stabilization of duplex formation (59). Modified oligonucleotides are usually synthesized using the “gapmer” approach, which consists of the core DNA sequence flanked by LNA sequences to achieve the necessary stability (60).

Recently, some experimental studies have established the ability of Stem 2 of the SARS-CoV-2 pseudoknot to act as a target site that can be utilized by ASOs for sequestration, as summarized in Table 2 (61). ASOs modified by both LNAs and guanidinium-type G-clamps (a cytosine analog that can form extra hydrogen bonds with guanine) were shown to be capable of fully destabilizing the pseudoknot at nanomolar concentrations (62). Meanwhile, ASO-LNA-DNA gapmers directed against the ORF1b region led to a decrease in the number of SARS-CoV-2 genomic copies by 71% in human cells (63). A combinatorial approach employing multiple LNA gapmers resulted in a reduction of intracellular viral loads by up to 96% in *in vitro* experiments (64).

**TABLE 2:** Antisense oligonucleotide-based strategies targeting the SARS-CoV-2 frameshift-stimulatory element

ASO strategy	RNA target	Experimental model	Activity/potency	Mechanism of action	Reference
LNA/G-clamp phosphorothioate ASOs	Stem 2 of the SARS-CoV-2 frameshift-stimulatory pseudoknot	*In vitro* structural analysis and antiviral assay	Nanomolar activity; inhibited viral replication	Destabilization of the pseudoknot structure, reducing −1 PRF	(58)
LNA-DNA gapmer ASOs	ORF1b	Human cell culture	Reduced SARS-CoV-2 genomic RNA copies by 71%	RNase H-mediated degradation of viral RNA	(59)
Multiple LNA gapmers	Conserved SARS-CoV-2 RNA genome	Cell culture	Reduced intracellular viral RNA by up to 96%	Sequence-specific degradation of viral RNA	(64)
LNA-modified ASO	SARS-CoV-2 RNA	K18-hACE2 mouse model	Suppressed lung viral replication by 98%–99% with an 80-fold reduction in viral titers	Sequence-specific inhibition of viral RNA	(65)

#### Structure-guided design and tertiary recognition

Insight provided by high-resolution structural information obtained through cryo-EM has enabled a transition from ASO design based on sequence to structure-based ASO design. According to the data obtained through cryo-EM analysis, the frameshift-stimulating element region of the SARS-CoV-2 virus is structured into a compact 28 kDa structure that includes three stems with the characteristic feature of threading of the 5′ end through the middle junction site. Knockdown of the virus was achieved at 100 nM concentration when targeting this threading region and the pseudoknot core (66).

Apart from the conventional method of linear hybridization, there is a design of “3D-ASOs,” which was designed to target the tertiary structure and hydrogen-bonding system of the pseudoknot. These compounds utilize structure-driven template designs to help in recognizing RNA structures with higher affinity, including Hoogsteen base pairing (67).

In addition, contemporary studies have shown that designing against specific structural motifs within RNA, such as the 5′ leader sequence or various types of stems, can shift the balance toward non-productive states that cannot accommodate ribosomal frameshifting (65).

#### Mechanistic pathways and translational efficacy

ASO inhibition of viral replication through frameshift-stimulating elements mainly occurs through two different mechanisms: RNase H-mediated degradation and steric inhibition. The mechanism involving RNase H involves the formation of RNA/DNA heteroduplexes via gapmers based on DNA, which recruits the endogenous enzyme RNase H, causing cleavage of the viral genome, thus ultimately resulting in the permanent loss of the frameshift-stimulating element (63). However, the sterically inhibitory ASOs, which usually use 2′-O-methyl or 2′-O-methoxyethyl modifications, inhibit the process through physical interference with ribosomal movement and translation machinery assembly without inducing transcript degradation (68).

The translational utility of these compounds has been further enhanced by recent *in vivo* studies. In K18-hACE2 mice, inhalation of LNA-modified ASOs led to a significant (98%–99%) suppression of viral replication in the lungs, resulting in an 80-fold reduction in viral titers (65). This targeted delivery route offers a significant advantage in the treatment of acute coronavirus infection, as it allows for the delivery of high concentrations of therapeutics into the respiratory epithelial tissue while reducing their systemic exposure (67).

Notably, due to the high degree of conservation of the frameshift-stimulating element sequences among the betacoronaviruses, ASOs have retained their potent activity against several variants of interest, such as the Delta and Omicron strains (65).

### Peptide nucleic acids

The PNA molecule is an artificial analog of the natural nucleic acid, and its structure is very different from the usual ASO. The natural phosphate-sugar backbone in PNAs is substituted with the peptide-like backbone composed of N-(2-aminoethyl)glycine residues (69). Such a backbone is nonionic and achiral, which is the unique structural characteristic of PNAs; thus, there are no electrostatic interactions between two strands, which increases the affinity and thermal stability of the hybridization process for RNAs compared with those for DNA or RNA molecules (70). Furthermore, the non-naturally occurring peptide-like bonds provide great biochemical resistance to degradation by nucleases (DNase I and S1 nuclease) or proteases (proteinase K), resulting in prolonged biological half-lives within the cellular environment (71). With respect to the drawbacks of earlier PNAs, including their inability to dissolve in water, along with the tendency to aggregate, a number of modifications have been introduced to overcome these issues (72, 73). This includes the use of γPNA, where modifications are made by introducing various groups at the γ-position of the N-(2-aminoethyl) glycine backbone, thereby improving the performance of PNA significantly. These modifications help achieve an initial organization of the molecule in a right-handed helical conformation that fits the geometry of the targeted RNA molecule (70). Modifications, such as the introduction of mini-PEG side groups, improve physicochemical characteristics (74). In doing so, preorganization lowers the entropic penalty incurred by binding, thus improving affinity and specificity (75). Chiral γ-PNAs exhibit better binding to native RNA than nonchiral ones (76). This preorganization makes γ-PNAs particularly good at recognizing highly organized sequences in viral genomic structures (77).

The unique physicochemical properties of PNA enable them to effectively destabilize RNA structural elements that regulate the −1 PRF process. Specifically, PNAs exert their functions through two main mechanisms: strand invasion and triplex formation (78). Due to their strong affinity, PNAs can enter an existing RNA double helix and kick out the complementary RNA strand to create a new PNA-RNA double helix, thus unfolding RNA pseudoknot structures suitable for frameshift regulation (79). In some instances, especially when dealing with homopurine sequences, PNAs are capable of triplex-forming through the Hoogsteen base pairing mechanism (80). These triplexes, composed of PNA-RNA-PNA, are highly stable, thus making it difficult for ribosomal frameshifting (81). In order to achieve formation at physiological pH levels, modified nucleobases such as thio-pseudoisocytosine (sJ) are employed, eliminating the necessity for protonation of cytosine but retaining its strong affinity to RNA duplexes (78, 82).

Antisense PNA activity against coronaviruses was evidenced experimentally; for instance, antisense PNA conjugates with cell-penetrating peptides have been shown to inhibit the SARS-CoV –1 PRF pseudoknot and, thereby, reduce frameshifting up to 94%. This frameshifting suppression leads to lower expression of viral replicase proteins and reduced viral titers *in vitro* (83). Recent studies on SARS-CoV-2 frameshift-stimulating element and its inhibition by antisense PNAs have further highlighted the importance of this mechanism in the viral life cycle. Experiments demonstrated that antisense PNAs targeting Stem 2 were more efficient in inhibiting frameshifting than those targeting Stem 1 and the slippery site (81).

It is worth noting that while complex PNA strategies like tail-clamps and bis-PNAs have been less effective in blocking the frameshift-stimulating element compared to straightforward antisense PNAs (81), the former can potentially produce highly thermodynamically stable triple helices. However, the kinetic accessibility may play a more critical role since antisense PNAs might be able to better access the structured RNA before the ribosome passes through the sequence (84). This highlights the importance of attacking the stem regions and junctions in the pseudoknot to inhibit the necessary RNA-ribosome interactions in order to cause stalling.

Although there are several advantages of using PNAs in pharmacology, there is still one major drawback in the application of these molecules. The neutral nature of the PNA backbone renders them unable to undergo passive diffusion through the lipid bilayer (70).

Some current strategies that have been implemented to overcome this problem include the conjugation of PNA molecules with CPPs (73) and the use of nanoparticulate delivery systems (85). In addition, the use of ligands such as 2-aminopyridine (M) in PNA sequences has been studied as an approach toward improving the molecular recognition of dsRNA (74).

PNA inhibitors that show high selectivity along with appropriate potency to qualify as potential candidates in the treatment of viruses associated with −1 PRF have been discovered. Combining high structural stability with high sequence selectivity, PNAs represent a potential approach for targeting −1 PRF-associated conserved RNA structures (83).

### RNA-targeting chimeras

RNA-targeting chimeras are hetero-bifunctional compounds designed to harness the innate RNA degradation pathways against specific transcripts. Unlike ASOs and PNAs that utilize stoichiometry and steric hindrance to inhibit target sites, RIBOTACs use a catalytic strategy. They act to attract cellular nucleases to the target site, allowing for the repetitive degradation of many RNA molecules through one therapeutic compound (86). The catalytic property is what makes RIBOTACs distinct from other occupancy-based inhibitors, and it could be used to improve antiviral activity at reduced doses (87).

In terms of structure, a RIBOTAC is composed of an RNA-binding module attached by a flexible linker to a recruiter module, often an inducer of ribonuclease L (88). Human ribonuclease L is a fundamental element of innate immunity and is usually present as an inactive protein until its dimerization, which is triggered by the presence of 2′−5′ oligoadenylates (89, 90). The utilization of a small-molecule inducer of RNase L as a recruiter module in RIBOTACs results in the induction of localized nuclease activity around the targeted RNA sequence (86). The close vicinity allows the protein to gain access to the RNA and break it down, thus converting it from a structural binder into an RNA-degrading molecule (5).

This catalytic approach has particular benefits when it comes to blocking the −1 PRF process. The ligand molecules designed on the principle of occupancy have to compete with either the fast RNA conformational changes or the physical force applied by the translating ribosomes, while RIBOTACs focus on the total elimination of the frameshift-stimulating element. The destruction mechanism allows avoiding difficulties associated with the complexity of the structures formed by the viral pseudoknots, often existing in several energetically favorable states (86). As a result of their action, the RIBOTAC molecules initiate the enzymatic degradation of the RNA structures responsible for the translation process (46).

The effectiveness of this technique was shown in studies involving the identification of oncogenic and disease-related RNA structures (91). Early studies showed that inactive small-molecule binders can be developed as potent RIBOTACs able to degrade the selected RNA selectively in cell culture. For example, Tong et al. (92) identified a small molecule that targets the asymmetric A-bulge in the cleavage site of Dicer for miR-155 precursors. While the starting molecule showed moderate micromolar binding affinity ($K_{d}\approx 2.2\;\mu M$), development of a RIBOTAC derivative allowed the selective destruction of the target RNA *in vitro* at a dose-dependent manner. Importantly, the chimeric compound selectively inhibited RNAs containing mutations in the target sites, which confirmed the exceptional specificity of the nuclease recruitment approach. Likewise, Costales et al. (93, 94) improved their RIBOTACs for targeting pre-miR-21 and pre-miR-210, showing that the inactivation of these two RNAs leads to decreased metastatic capability of cancer cells *in vivo*.

The method has proved successful in the case of the SARS-CoV-2 genome, providing a blueprint for the development of drugs against coronavirus. Haniff et al. (46) have discovered a small molecule with pharmaceutical properties that targets the 1 × 1 UU nucleotide internal loop of the attenuator hairpin located on the frameshift-stimulating element of SARS-CoV-2 (95). The interaction between the small molecule and the target is measured by the dissociation constant (*K*_*D*_) value of 11 nM. While the binding agent stabilizes the hairpin structure and inhibits frameshifting, the bioactivity improves by 10-fold when converted to C5-RIBOTAC (Table 3) (46). This chimera molecule effectively recruited RNase L to bind to the viral RNA, enabling the direct degradation of RNA molecules with a dramatic decrease in RNA load compared to that achieved by only binding (46, 95). For the verification of the interaction with the target, scientists used a modified, covalent form of the molecule called C5-Chem-CLIP (46).

**TABLE 3:** RNA-targeting chimeras directed against the SARS-CoV-2 frameshift-stimulatory element

RNA-targeting chimera	RNA target	Experimental model	Activity/potency	Mechanism of action	Reference
C5-RIBOTAC	1 × 1 UU internal loop of the attenuator hairpin within the SARS-CoV-2 frameshift-stimulatory element	Biochemical and cellular assays	*K*_*D*_ = 11 ± 3.8 nM (parent ligand C5); ~10-fold increase in bioactivity following conversion to C5-RIBOTAC	Recruitment of endogenous RNase L to promote degradation of the FSE, resulting in reduced viral RNA load	(46, 95)
Bio-orthogonal RIBOTACs	SARS-CoV-2 frameshift-stimulatory element	Cellular assays	Inducible RNA degradation; quantitative potency not reported	Bio-orthogonal (“caged”) recruitment of RNase L for stimulus-dependent degradation of viral RNA	(88)

Recent literature has expanded on this system through the engineering of bio-orthogonal RIBOTACs for inducible degradation. Xiong et al. (88) described a SARS-CoV-2-targeted RIBOTAC equipped with a “caged” moiety that prevents RNase L binding in the absence of an activating signal. The inducible nature of this design allows for greater control over RNA degradation, providing a controllable and effective platform with practical prospects for addressing potential viral pandemics.

While there has been advancement in this field, the clinical translation of RIBOTACs will require fine-tuning of various chemical properties. Target specificity is dependent on both the type and size of the linker, which should be able to properly position RNase L relative to the target sequence without hindering the interaction between the recognition module (96). In addition, transcriptome-wide selectivity is essential since non-specific targeting of RNase L to undesired RNAs may result in severe toxicity (96, 97). Recent studies have focused on unbiased screening methods that can help determine factors that contribute to specificity, such as the proximity of favored RNase L motifs (UA or UU dinucleotides) to the small molecule binding site (96, 98).

In summary, RNA-based chimeric molecules represent a viable strategy for the management of dynamic and complex RNA motifs associated with viruses. The use of cellular endonucleases by RIBOTACs can provide suppression of −1 PRF, without the need for extensive structural changes in the target pseudoknot motif (46). Continued developments in medicinal chemistry and RNA structure elucidation are expected to position RIBOTACs as an emerging platform that warrants further evaluation for targeting viral RNAs (46, 88).

## CHALLENGES AND FUTURE PERSPECTIVES

Despite significant advances in understanding the structural organization and conformational dynamics of coronavirus frameshifting pseudoknots, translating these findings into effective antiviral therapies remains a significant challenge. Most pseudoknot-targeting compounds have been evaluated using reporter assays, biochemical experiments, or cellular models, whereas relatively few studies have examined their antiviral activity in the context of viral infection. Only a limited number of studies have demonstrated inhibition of authentic SARS-CoV-2 replication, emphasizing the need for further validation and optimization in relevant preclinical models. Furthermore, the antiviral effects reported so far are generally modest, highlighting the need for improved potency, selectivity, and validation in physiologically relevant systems. Consequently, the SARS-CoV-2 frameshift-stimulatory pseudoknot should currently be regarded as a potential antiviral target that requires further preclinical investigation.

The inherent structural flexibility of the pseudoknot presents additional challenges to therapeutic development. Experimental and computational studies have demonstrated that the frameshift-stimulatory element exists as a dynamic ensemble of interconverting conformations rather than a single stable structure. Although this conformational diversity creates opportunities for RNA-targeted intervention, it also complicates ligand design because compounds must selectively stabilize or modulate functionally relevant conformations with sufficient affinity and specificity. Achieving high selectivity is particularly challenging because related structural motifs are also found in cellular RNAs, increasing the risk of off-target interactions.

Continued progress will depend on the development of RNA-targeting molecules with improved potency, selectivity, and pharmacological properties, together with their evaluation in biologically relevant infection models. Structure-guided drug design, molecular dynamics simulations, and ensemble-based screening are expected to facilitate the identification of ligands that recognize functionally important pseudoknot conformations. For ASOs and PNAs, advances in chemical modification and delivery technologies are likely to improve intracellular stability and target engagement, whereas RIBOTAC-based approaches require further evaluation of their specificity, efficacy, and safety. Ultimately, validation in viral replication systems and appropriate animal models will be essential to determine whether modulation of programmed −1 ribosomal frameshifting can be translated into a clinically useful antiviral strategy.
